# CRITIC–EDAS Approach for Evaluating Mechanical Properties of Flax/Vetiver/MFF Hybrid Composites

**DOI:** 10.3390/polym17131790

**Published:** 2025-06-27

**Authors:** M. Navin, Thirumalaisamy Ramakrishnan, Devarajan Balaji, Venkateswaran Bhuvaneswari

**Affiliations:** 1 Department of Mechanical Engineering, CMS College of Engineering and Technology, Coimbatore 641032, Tamil Nadu, India; 2 Department of Mechanical Engineering, Sri Eshwar College of Engineering, Coimbatore 641202, Tamil Nadu, India; ramakrishnan.t@sece.ac.in; 3 Department of Mechanical Engineering, KPR Institute of Engineering and Technology, Coimbatore 641407, Tamil Nadu, India; balaji.ntu@gmail.com (D.B.); bhuvanashankar82@gmail.com (V.B.); 4 AU-Sophisticated Testing and Instrumentation Centre, Department of Mechanical Engineering, Alliance School of Applied Engineering, Alliance University, Bengaluru 562106, Karnataka, India

**Keywords:** hybrid composites, flax fibers, vetiver fibers, mahogany fruit filler (MFF), tensile strength, flexural strength, impact resistance, CRITIC–EDAS method

## Abstract

This study investigates the mechanical properties and optimization of hybrid composites composed of flax, vetiver, and mahogany fruit fillers (MFFs) using epoxy resin as the matrix material. Nine distinct composite configurations were fabricated using different MFF concentrations (0, 5, and 10 wt.%) to evaluate their influence on tensile strength, flexural strength, and impact resistance. The MFF was subjected to alkali treatment and characterized using FTIR, XRD, and particle size analysis to enhance its compatibility with the polymer matrix. Vetiver and flax fibers also underwent alkali treatment to improve interfacial bonding. The composite fabrication process followed the Taguchi L9 orthogonal array to optimize the design. Mechanical testing revealed that the incorporation of MFF significantly improved the overall performance, with FVM9 (10 wt.% MFF) exhibiting the highest tensile strength (56.32 MPa), flexural strength (89.65 MPa), and impact resistance (10.46 kJ/m^2^). The CRITIC–EDAS method was employed to rank the composite configurations, and FVM9 was identified as the optimal configuration. Comparisons with alternative MCDM methods (WASPAS, COPRAS, TOPSIS, and VIKOR) validated the reliability of the rankings, and FVM9 consistently performed the best. The sensitivity analysis demonstrated the robustness of the CRITIC–EDAS approach, as the rankings remained stable despite variations in the criterion weights. The synergistic effect of flax, vetiver, and MFF, along with improved interfacial bonding, contributed to the superior mechanical properties of the hybrid composites. These findings highlight the potential of FVM composites as sustainable, high-performance materials for various industrial applications in the automotive, construction, and aerospace sectors.

## 1. Introduction

Composite materials have transformed material science by offering a new path that combines multiple constituents to develop rigid structures with enhanced and tailored properties [1]. Specifically, natural fiber composites, with their lightweight design, mechanical strength, and ecological friendliness, are considered the most sustainable alternatives to synthetic materials [2,3,4,5]. Furthermore, hybrid composites have reached the next level of combining fibers or fillers with different material strengths, making them suitable for a wide range of industrial applications, including automotive (e.g., interior panels and dashboards), construction (e.g., lightweight structural components and insulation boards), aerospace (e.g., non-structural interior parts), and marine industries (e.g., boat interiors and decking materials) [6,7,8,9]. Current trends in natural fibers in different parts of the globe skew towards environmentally friendly solutions and the utilization of renewable resources. Among the available natural fibers, flax has been of considerable interest owing to its remarkable tensile strength, biodegradability, and low density, which make it a preferred choice for structural reinforcements [10,11]. Rajesh et al. [12] showed that flax fibers have the potential to increase the tensile strength and stability of composites, making them significant in material design. Similarly, vetiver fibers are recognized for their exceptional energy absorption capabilities and thermal stability, making them indispensable in applications involving dynamic loading [13,14]. Similarly, Alipour et al. [15] observed a 20–26% increase in the tensile strength of fine twill flax/epoxy composites compared to coarse twill, with unidirectional fabrics showing a tensile strength of up to 239 MPa (*p* < 0.05). Hadj-Djilani et al. [16] found that angle-ply flax/epoxy laminates exhibited 3.5–19.11% higher impact resistance than cross-ply and quasi-isotropic configurations, with post-impact flexural strength declining by 14–26%. Although flax fiber as a single reinforcement in polymer composites achieves moderate strength, it is surpassed by hybrid composites with synergistic reinforcement. For instance, Graupner et al. [17] demonstrated that flax/glass hybrid composites exhibited approximately 20–22% higher impact strength than single-fiber flax composites, which was attributed to the enhanced damage tolerance from the synergy of glass fibers. Similarly, Calabrese et al. [18] reported improved mechanical stability and impact performance of flax/glass hybrids under environmental stress, reinforcing their superiority over single-fiber flax composites.

In particular, when hybridized with root fibers, flax-reinforced composites exhibited better mechanical performance. For instance, Krishnakumari et al. [19] investigated flax (bast fiber) and vetiver (root fiber) hybrid composites in a polyester matrix, reporting a 15–20% increase in impact strength over single-fiber flax composites due to vetiver’s superior energy absorption. Similarly, Vinay et al. [20] found that flax/vetiver hybrids exhibited an 18% improvement in flexural strength, which was attributed to enhanced fiber–matrix bonding and vetiver structural stability. These studies indicate that additional reinforcement is necessary to further enhance the mechanical properties of flax-based composites. Janyakunmongkol et al. [21] emphasized vetiver’s role by introducing filler content, which significantly improved tensile, compressive, and flexural properties up to an optimal point (5 to 10 wt.%). Tensile strength increased from 7.86 to 27.47 MPa as mahogany fruit filler (MFF) loading increased from 10% to 30% [22]. The filler probably contained high levels of cellulose (75.61%), which likely acted as a reinforcing agent, helping the composite carry more load and resist deformation, thus increasing the tensile and flexural strength [23]. Compressive strength increased from 14.54 to 48.19 MPa when the MFF content increased from 10% to 30% [24]. Flexural strength improved from 33.44 to 67.65 MPa as the filler content increased from 20% to 40% [25]. Studies [26,27,28] have also reported improvements in Young’s modulus, strain, and energy absorption in various mechanical tests. However, these properties may decline at excessive filler loadings (>10 wt.%). This underscores the potential of simultaneously incorporating these fibers into a single matrix to produce multifunctional materials with enhanced performance. However, it is difficult to determine the optimal composition for a specific application. Hence, recent studies have focused primarily on mechanical testing, with minimal application of advanced decision-making frameworks [29,30,31,32,33], to optimize hybrid composite compositions.

For example, Saha et al. [34] explored the mechanical and thermal properties of *Bambusa Tulda* biocomposites reinforced with various epoxy matrices. Their findings revealed that composites with 30% bamboo fiber exhibited tensile strengths of up to 144.76 MPa and a flexural modulus of 8.42 GPa. S2 composites have emerged as the most suitable materials for automotive applications, owing to their superior tensile strengths and minimal moisture absorption. Using the VIKOR method, the study successfully identified optimal configurations and highlighted challenges, such as hydrophilicity and weak interfacial bonding, that require further optimization. Similarly, Chandrika et al. [35] studied hybrid composites composed of kenaf fibers and varying percentages of sawdust. The incorporation of 5 wt.% sawdust improved tensile strength by 3.34 times and increased flexural strength by 32%, compared to sawdust-free composites. However, the impact decreased owing to reduced elasticity. Using the multi-attribute decision-making (MADM)–VIKOR method, it was found that the 5 wt.% configuration is the most balanced in terms of mechanical performance and density. This demonstrates the stability of agricultural waste, which can enhance composite properties and maintain cost-effectiveness.

Mahajan et al. [36] utilized the CRITIC and TOPSIS methods to evaluate and rank natural fibers for sustainable composite development. By comparing fibers such as basalt, hemp, and kenaf, this study identified basalt as the most effective material, owing to its high tensile modulus and cost efficiency. The sensitivity analysis further validated the consistent performance of basalt under various criteria weights, confirming its suitability for automotive and medical applications. Mahesh et al. [37] explored the potential of rubber crumb as a filler in kenaf-based hybrid composites. They found that incorporating 3 wt.% rubber crumb increased tensile strength by 24.5% and flexural strength by 36.83%, while 5 wt.% rubber crumb reduced water absorption by 2.4 times compared to unfilled composites. This study employed the VIKOR method to identify the 5 wt.% configuration as optimal, noting a 54.33% improvement in impact resistance. This study highlighted the importance of integrating waste materials to enhance mechanical and moisture-resistant properties.

Mahesh et al. [38] evaluated polylactic acid (PLA) biocomposites reinforced with wood and rice husk waste. Their findings showed that composites containing 7.5 wt.% wood waste PLA exhibited the highest tensile modulus of 4.24 GPa. However, increasing the rice husk content led to higher water absorption, presenting a trade-off between mechanical performance and durability. The hybrid CRITIC–Multi-Attributive Border Approximation Area Comparison (MABAC) approach employed in this study effectively ranked composite configurations, offering a comprehensive framework for the utilization of agricultural waste in biopolymers. Similarly, Sriariyanun et al. [39] investigated hybrid composites made from Borassus fruit fibers and synthetic cellulose acetate augmented with eggshell powder. The incorporation of 5 wt.% eggshell powder enhanced tensile strengths to 53.62 MPa and impact strengths to 38.67 MPa. Using the CRITIC–EDAS method, the 5 wt.% configuration was deemed optimal, demonstrating consistent rankings through sensitivity analysis, emphasizing the utility of bio-fillers for sustainable and cost-effective composites. These studies demonstrate that multi-criteria decision-making (MCDM) methods, such as CRITIC and EDAS, offer significant potential for material selection and optimization. However, their application in flax/vetiver/mahogany (FVM)-based hybrid composites has not yet been explored. This dual gap in material combinations and methodological approaches highlights the need for robust and systematic investigations.

Although independent studies have examined flax for its tensile properties and vetiver for impact absorption, the combined use of these fibers with natural filler reinforcements, such as MFF, in hybrid composites has not yet been extensively explored. The present study addresses these gaps by fabricating and analyzing hybrid composites composed of flax, vetiver, and MFF, using epoxy as the matrix material. Nine distinct composite configurations (FVM1–FVM9) are developed with varying mahogany fruit filler (MFF) concentrations (0, 5, and 10 wt.%) combined with flax and vetiver fibers (10, 15, and 20 wt.% each) to evaluate their influence on mechanical performance. Mechanical properties, including tensile strength, flexural strength, and impact resistance, are systematically tested, as they are critical determinants for structural applications. In addition, the CRITIC–EDAS method is employed to rank the composite configurations, thereby providing a data-driven framework for identifying the optimal combination. By integrating experimental testing with advanced decision-making techniques, this study offers a comprehensive evaluation of FVM hybrid composites and contributes significantly to the growing field of sustainable material research. In addition, this study compares the optimized filler concentration with alternative MCDM methods, such as WASPAS [33], COPRAS [40], TOPSIS [41], and VIKOR [29,35], to validate the findings. This comprehensive methodology ensures reliable rankings while offering valuable insights into the most effective composite configuration.

## 2. Materials and Methods

### 2.1. Materials

Mahogany fruits and vetiver roots were obtained from Coimbatore (Tamil Nadu, India). A unidirectional flax fiber mat was obtained from Go-Green Composites (Chennai, Tamil Nadu, India). Epoxy resin (LY 556), hardener (HY 951), and 99% pure NaOH pellets were provided by Covai Seenu & Co. Ltd. (Coimbatore, Tamil Nadu, India).

### 2.2. Preparation of Mahogany Fruit Fillers (MFFs)

**Figure 1:**
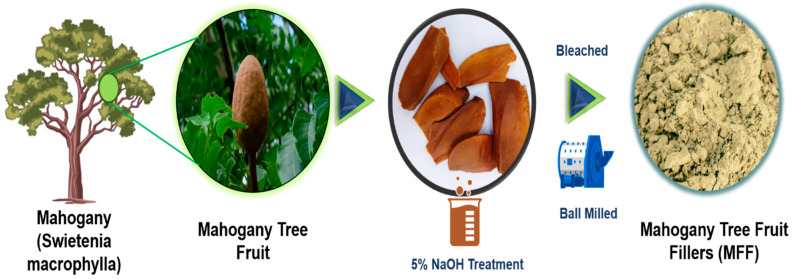
Isolation of mahogany fruit fillers (MFFs).

Mahogany fruit (***Family*:** *Meliaceae*; ***Order*****:** *Sapindales*) of the genus *Swietenia* was carefully processed to remove the seeds and outer shell, exposing the fibrous core rich in cellulose (40–50%) and lignin (20–35%), as listed in Table 1. The extracted core was cut into smaller pieces to facilitate further processing (Figure 1). To enhance its compatibility with the polymer matrix, mahogany fruit fiber was subjected to alkali treatment using a 5% NaOH solution. This treatment effectively removed impurities, hemicellulose, and lignin to improve interfacial adhesion, as shown in Table 1. After the alkali treatment, the fibers were thoroughly washed with distilled water to eliminate any residual NaOH and subsequently dried in a hot air oven at 60 °C for 24 h to remove any remaining moisture. Once dried, the fibers were ground into a fine powder using a mechanical grinder and sieved to achieve particle sizes ranging from 30 to 50 µm. The refined MFF was then incorporated as a reinforcing material into the composite fabrication process, contributing to the improved mechanical performance and sustainability of the composites.

#### 2.2.1. FTIR Analysis of Untreated and Treated Mahogany Fruit Fillers (MFFs)

**Figure 2:**
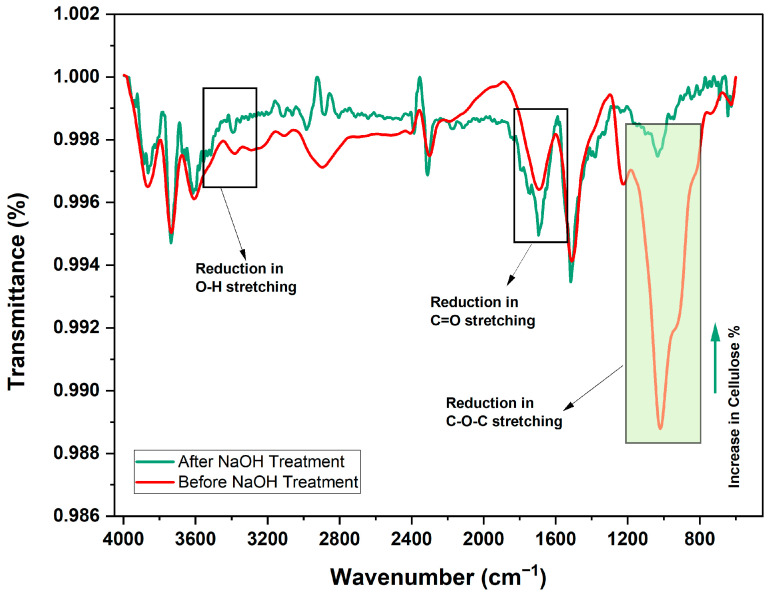
FTIR of mahogany fruit fillers (MFFs).

The FTIR analysis (***Make*:** FT/IR-4000 Series—Jasco Inc., Tokyo, Japan) of untreated and 5% NaOH-treated mahogany fruit fillers (MFFs) across the 4000 to 400 cm^−1^ wavenumber range shown in Figure 2 revealed significant chemical changes that enhanced the mechanical strength of the composites. The spectra show a reduction in O-H stretching (3400 cm^−1^) and C=O stretching (1730 cm^−1^) peaks after treatment, indicating decreased hemicellulose (18.2% to 10.3%) and lignin (23.5% to 13.1%) contents, alongside a lower moisture content (11.1% to 7.6%). Cellulose-related C-O-C peaks (1050 cm^−1^) became more prominent, reflecting an increase in cellulose content (37.4% to 52.5%). These changes improved the mechanical properties of the MFF-reinforced composites by enhancing cellulose reinforcement, which increased the tensile strength of the natural filler hybrid composites and increased fiber–matrix bonding through reduced amorphous components, leading to a 15–20% improvement in tensile and flexural strength. Additionally, the reduced moisture content improves dimensional stability, ensuring consistent performance under load, making MFF a promising filler for high-strength composite applications.

#### 2.2.2. XRD Analysis of Untreated and Treated Mahogany Fruit Fillers (MFFs)

**Figure 3:**
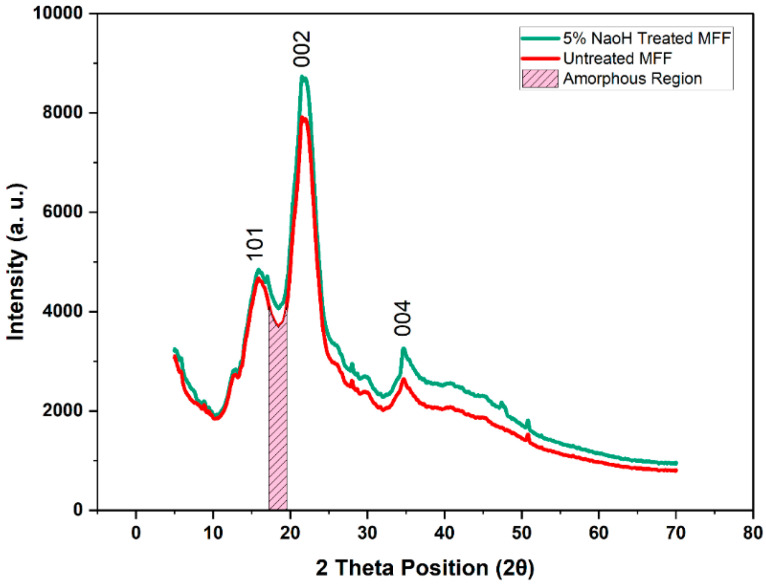
XRD of mahogany fruit fillers (MFFs).

XRD analysis (Figure 3) of untreated and 5% NaOH-treated mahogany fruit fillers (MFFs) in the 2θ range of 0° to 80° highlights the structural enhancements that significantly improve the mechanical properties of MFF-reinforced composites. The diffractograms show characteristic peaks of cellulose I, identified by the (101), (002), and (004) planes at 2θ = 15.96°, 21.57°, and 34.21° for untreated MFF, which shifted slightly to 16.09°, 21.87°, and 34.67° post treatment, with the (002) peak intensity indicating higher crystallinity. Cellulose I, the native form of cellulose in plants, was confirmed by these peak positions, which are typical of its monoclinic lattice structure and are commonly found in lignocellulosic materials such as mahogany. The amorphous region is identified by the minimum intensity between the (101) and (002) peaks at 2θ = 18.61°, with intensities of 1000 a.u. for untreated MFF and 800 a.u. for treated MFF, reflecting non-crystalline components such as hemicellulose and lignin. The crystallinity index (CrI), calculated using the Segal method, increased from 86.67% to 90.07%, driven by the removal of amorphous hemicellulose (18.2% to 10.3%) and lignin (23.5% to 13.1%), while the cellulose content increased from 37.4% to 52.5%. The crystal size, determined using the Scherrer equation, increased from 8.10 to 8.99 nm, indicating larger crystallites and improved structural order. These changes enhance the composite tensile strength by increasing cellulose reinforcement and stiffness, while improving fiber–matrix bonding due to reduced amorphous content, boosting the tensile and flexural strengths. Additionally, the lower moisture content (11.00% to 7.00%) inferred from the reduced amorphous regions ensured dimensional stability, supporting consistent mechanical performance under load, making treated MFF a promising filler for high-strength composite applications.

#### 2.2.3. Particle Size Analysis of Treated Mahogany Fruit Fillers (MFFs)

**Figure 4:**
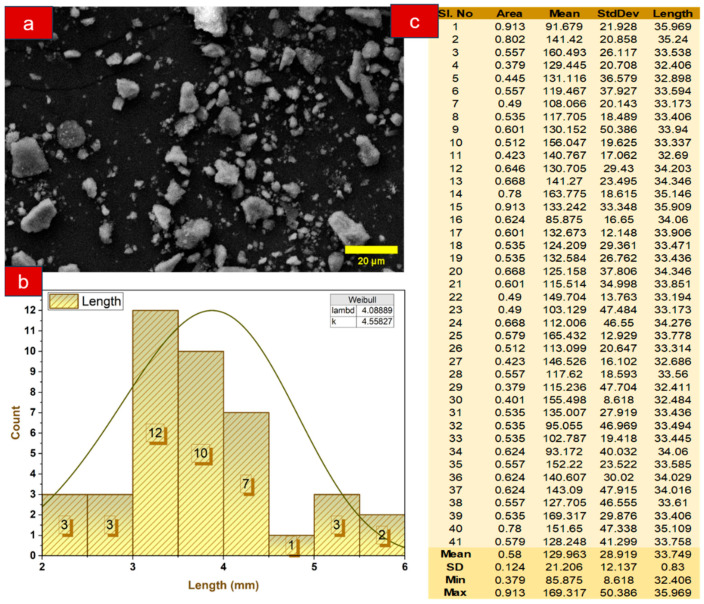
Particle size analysis of mahogany fruit fillers (MFFs): (**a**) SEM of MFFs; (**b**) particle size distribution curve; and (**c**) particle size distribution data.

Particle size analysis (Figure 4) of 5% NaOH-treated mahogany fruit fillers (MFFs), conducted using ImageJ software (Version: 1.54p) on scanning electron microscopy (SEM) images, provided insights into the refined particle size distribution and its role in enhancing the mechanical properties of MFF-reinforced composites. The SEM images were processed using ImageJ by converting them to 8-bit grayscale, applying a threshold to isolate the particles, and using the “Analyze Particles” function, revealing a mean particle size of 33.749 µm with a standard deviation of 0.83. The analysis showed that a significant portion of the particles was below 40 µm, a result of alkali treatment removing hemicellulose and lignin, which causes fibrillation and breaks down fiber bundles into finer fragments, as observed in the SEM images displaying increased surface roughness and smaller particle clusters. This reduction in particle size increases the surface area, improving fiber–matrix interfacial bonding in the composites by enhancing adhesion and load transfer, which contribute to higher tensile and flexural strengths. Finer particles also ensured better dispersion within the polymer matrix, reducing voids and promoting uniform reinforcement, leading to improved mechanical consistency across the composite.

### 2.3. Preparation of Vetiver Grass and Flax Fiber

**Figure 5:**
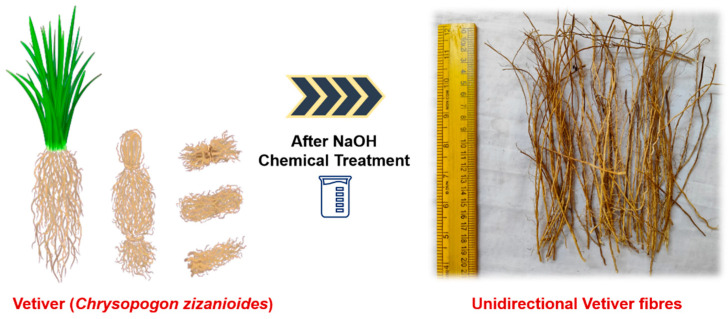
Preparation of unidirectional vetiver grass fibers.

Vetiver grass (*Vetiveria zizanioides*), renowned for its soil-stabilizing properties, is prevalent in the tropical regions of South India and is valued for its medicinal application. After serving the purpose of soil stabilization, vetiver grass is typically discarded as agro-waste. In this study, discarded vetiver agro-waste was collected, and the roots were separated and cleaned to remove the soil and debris. The cleaned roots were sun-dried before further processing (Figure 5).

Both vetiver and flax fibers underwent 5% NaOH treatment for 4 h, following protocols established in previous studies. After treatment, the fibers were thoroughly rinsed with distilled water to remove any residual NaOH. Finally, the treated fibers were dried in a hot air oven at 60 °C for 24 h to remove moisture from the samples. The chemical compositions of the vetiver and flax fibers before and after the 5% NaOH treatment are presented in Table 2.

### 2.4. Design of Experiments

Before fabricating the FVM hybrid composites, it is important to investigate the correlation between the control factors, particularly the filler concentration and fiber arrangement. Therefore, a design of experiment (DoE) was performed using the Taguchi approach, which is widely recognized for optimizing experimental processes and improving output accuracy [42,43,44,45]. It was also used to assess the influence of independent variables on achieving optimal MFF concentrations while minimizing the number of experimental runs required. Despite its efficiency, the Taguchi method provides a single optimal solution for each response, rendering it highly suitable for studies aimed at achieving specific performance improvements. In this study, the Taguchi design was applied to minimize the number of experimental trials while effectively analyzing the factors influencing the composite manufacturing process. Minitab 18 software was used to implement the Taguchi Design of Experiments (DoE). A Taguchi L9 (3 × 3) orthogonal array was used to identify the primary process factors and determine their optimal MFF concentrations. Table 3 and Table 4 present the process parameters, their respective levels, and the combinations used in the Taguchi design.

### 2.5. Fabrication of the Flax/Vetiver/MFF-Based Composites

**Figure 6:**
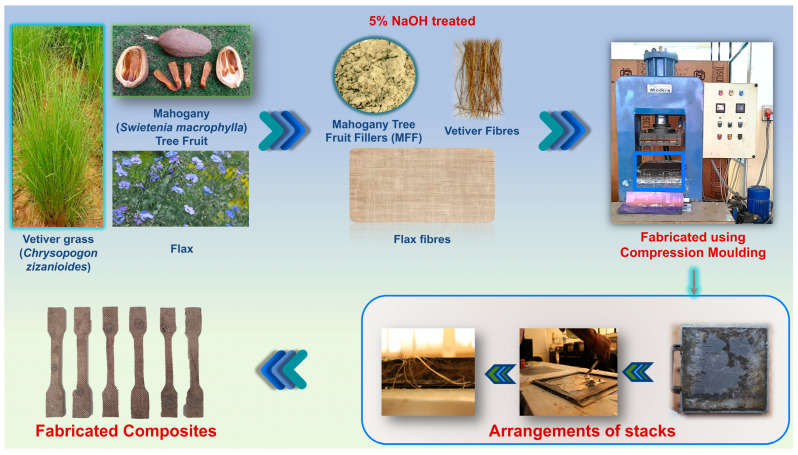
Fabrication of flax/vetiver/MFF hybrid composites.

Hybrid composite laminates comprising flax, vetiver, and MFF were fabricated with dimensions of 270 × 270 × 3 mm^3^ using a standard compression molding process following the Taguchi Design of Experiments (DoE) methodology to optimize the process parameters, as shown in Figure 6. The mold setup included a top die, bottom die, and middle square mold with a thickness of 3 mm. Initially, polyvinyl alcohol (PVA) was applied as a releasing agent to ensure the easy separation of the samples after fabrication. The fabrication process began with the preparation of a resin system. MFF was incorporated into the epoxy resin at concentrations of 0, 5, and 10 wt.%. The resin–filler mixture was stirred using a mechanical stirrer to achieve a uniform distribution and then combined with a hardener in a 10:1 ratio.

For the hybrid FVM composite, the process involved coating the mold surface with the prepared resin–filler mixture. A layer of flax fiber mat was placed over this coating, followed by another layer of the resin–filler mixture. This sequence was repeated until three flax fiber layers were stacked, and a final resin–filler layer was applied on top. The mold was closed with the top die, and a load of 750 N was applied to maintain a uniform pressure, resulting in a compressive pressure of 7.5 bar on the laminate. The vetiver root fibers were prepared by weighing them to match the weight of the three flax fiber layers. The unidirectional vetiver fibers were evenly arranged in the mold, and the resin–filler mixture was poured over them. A compressive force was applied to complete the fabrication of the composite. Post-curing was performed in a hot air oven at 80 °C for 8 h to remove impurities.

### 2.6. Testing of FVM Composites

The tensile test adhered to the ASTM D3039 standards [46] and was conducted using a Universal Testing Machine (UTM) (***Make:*** Zwick/Roell; ***Model:*** Z010, Kennesaw, GA, USA) at room temperature. To ensure accuracy, six replicates were tested for each FVM composite variant were tested. The flexural test was performed on a UTM at ambient temperature, following the ASTM D790 guidelines [47], with sample dimensions of 125 mm × 13 mm × 3 mm. Six sets of tests were conducted for each composition using a three-point bending method. For the impact tests, the ASTM D256 standard [48] was followed using an Izod notched testing machine. Test samples measuring 65 mm × 13 mm × 3 mm were subjected to six repetitions for each composite to ensure consistent and reliable results.

### 2.7. CRITIC Technique

**Figure 7:**
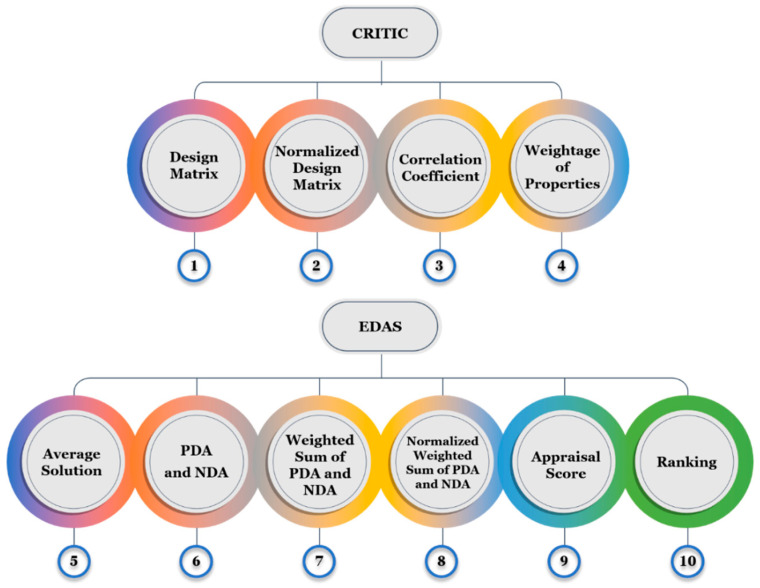
Computation flow of CRITIC and EDAS.

The Criteria Importance Through Inter-Criteria Correlation (CRITIC) method is a robust statistical technique used in MCDM to determine the relative importance of evaluation criteria. It leverages the variability of each criterion and the correlation between the criteria to assign objective weights, thereby ensuring a balance between the significance of individual properties and their interdependence. By analyzing contrast variation and inter-criteria conflict, the CRITIC method provides an unbiased assessment of criteria weightage, making it particularly effective for applications requiring systematic and data-driven decision-making. This method begins with the creation of a design matrix that includes all relevant criteria and their respective values, as illustrated in Figure 7. The matrix was then normalized to ensure that all the criteria were comparable and dimensionless. Subsequently, the correlation coefficient is calculated to measure the degree of interdependence between the criteria, highlighting their unique contributions to the decision-making process. Using these coefficients, the weight of each property was computed based on both contrast variability and intercriteria conflict. The CRITIC method effectively quantifies the importance of individual criteria, which is a critical input for ranking and optimization in subsequent analyses.

In this study, the CRITIC method was used to assign weights to various properties that influence the performance of the developed FVM hybrid composites, such as tensile strength, flexural strength, and impact resistance. These weights were incorporated into the EDAS method to evaluate and rank the composite variants based on their performance. This combined approach of CRITIC and EDAS ensured a systematic, objective, and reliable assessment of composite properties, enabling the identification of optimal configurations for specific applications. Detailed algorithms are provided in the Supplementary File.

### 2.8. EDAS Method

The Evaluation based on the Distance from Average Solution (EDAS) method is another MCDM approach that evaluates alternatives based on their proximity to an average solution. By calculating the Positive Distance from the Average (PDA) and Negative Distance from the Average (NDA), the EDAS determines how well each alternative performs compared to the average. This method is particularly effective for ranking alternatives because it combines weighted performance measures to produce a final appraisal score. The EDAS is known for its simplicity, reliability, and ability to handle multiple conflicting criteria, making it a popular choice in decision-making applications. The sequential steps followed in EDAS are provided in the Supplementary File.

## 3. Results and Discussions

The mechanical properties of the flax/vetiver/mahogany fruit filler (FVM) hybrid composites, evaluated across nine configurations (FVM1–FVM9), are intricately tied to the microstructural interactions between flax, vetiver, and MFF within the epoxy matrix. The following subsections provide a detailed analysis of the tensile, flexural, and impact properties, focusing on the cause-and-effect relationships at the microstructural level that lead to improvements or deterioration in performance.

### 3.1. Tensile Properties of Flax/Vetiver/MFF (FVM) Hybrid Composite

**Figure 8:**
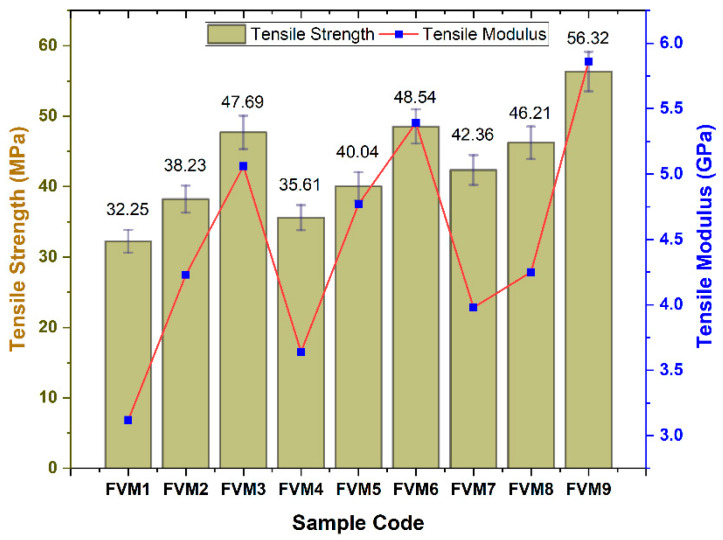
Tensile properties of flax/vetiver/MFF (FVM) hybrid composites.

The tensile strength of the FVM composites peaked at 56.32 MPa for FVM9 (20% flax, 20% vetiver, 0% MFF), whereas FVM1 (10% flax, 10% vetiver, 0% MFF) exhibited the lowest at 32.25 MPa, with tensile modulus values reaching 5.9 GPa for FVM9 (Figure 8). At the microstructural level, FVM9’s superior tensile strength is driven by the high cellulose content of flax fibers (73.7% post NaOH treatment), which enhances fiber stiffness and facilitates efficient load transfer along the fiber axis. The increased fiber content (20% flax, 20% vetiver) ensured a more uniform fiber distribution, minimizing stress concentrations and reducing void content, which is often a source of failure in natural fiber composites. Vetiver fibers, with 48.7% cellulose and higher flexibility due to 6.8% lignin, act as secondary reinforcements, bridging microcracks and distributing stresses across the matrix, as their rougher surface (post alkali treatment) enhances mechanical interlocking with the epoxy. The absence of MFF in FVM9 avoided potential agglomeration, which can occur at higher filler contents (>10 wt.%), leading to stress concentration points and reduced tensile strength, as observed in FVM3 (5% MFF, 41.87 MPa).

In contrast, FVM1’s lower fiber content (10% each) results in a sparse fiber network, increasing void formation and weakening interfacial bonding, as fewer fibers are available to bear tensile loads. Alkali treatment further improves tensile performance by reducing hemicellulose content (e.g., from 16.1% to 9.5% in flax), which minimizes hydrophilic sites and enhances fiber–matrix adhesion, as evidenced by FTIR analysis (reduced O-H stretching at 3400 cm^−1^). However, in configurations with MFF (e.g., FVM6, 48.76 MPa), the finer particles of filler (mean size 33.75 µm) fill the micro-voids, improving matrix cohesion, although excessive MFF can lead to particle clustering, slightly reducing tensile strength owing to the uneven stress distribution. This study aligns with previous studies that reported the optimal concentrations of filler materials [49,50,51].

### 3.2. Flexural Properties of Flax/Vetiver/MFF (FVM) Hybrid Composite

**Figure 9:**
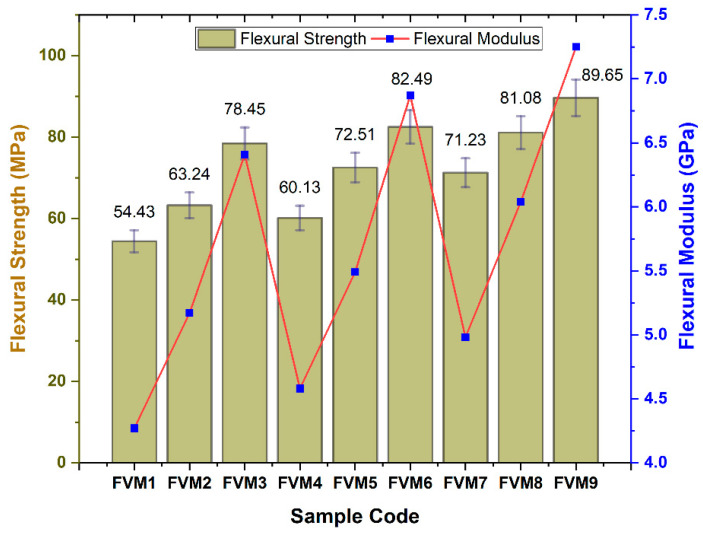
Flexural properties of flax/vetiver/MFF (FVM) hybrid composites.

The flexural strength was highest for FVM9 at 89.65 MPa, with a modulus of 7.0 GPa, whereas FVM1 recorded the lowest at 54.43 MPa (Figure 9). Microstructurally, FVM9’s enhanced flexural performance results from its layered configuration, where flax fibers form a rigid core (73.7% cellulose, high stiffness) and vetiver fibers, as outer layers, provide toughness (6.8% lignin). This arrangement minimizes shear stresses at the fiber–matrix interface during bending, as flax resists compressive forces on the inner layers, whereas vetiver’s flexibility absorbs tensile stresses on the outer layers, reducing the likelihood of delamination. The higher fiber content in FVM9 (20% each) ensured a denser fiber network, decreasing void content and enhancing stress transfer, as voids can act as crack initiation sites under flexural loading.

Alkali treatment further strengthened this by increasing surface roughness (e.g., the roughness of vetiver hemicellulose was reduced from 39.8% to 22.5%), improving interfacial adhesion, and preventing fiber pull-out, which is a common failure mode in natural fiber composites. In MFF-inclusive samples, such as FVM6 (82.49 MPa, 10% MFF), the high crystallinity of the filler (90.07% post treatment) enhances matrix stiffness by filling micro-gaps, thereby reducing void-induced crack propagation. However, FVM1’s lower fiber content (10% each) leads to a higher void fraction and weaker interfacial bonding, as the sparse fiber distribution fails to resist bending stresses, resulting in early delamination and crack growth at the microstructural level. The presence of MFF at higher concentrations (e.g., FVM3, 5% MFF, 62.31 MPa) can cause particle agglomeration, creating stress concentration points that initiate microcracks under flexural loading, further deteriorating performance.

### 3.3. Impact Properties of Flax/Vetiver/MFF (FVM) Hybrid Composites

**Figure 10:**
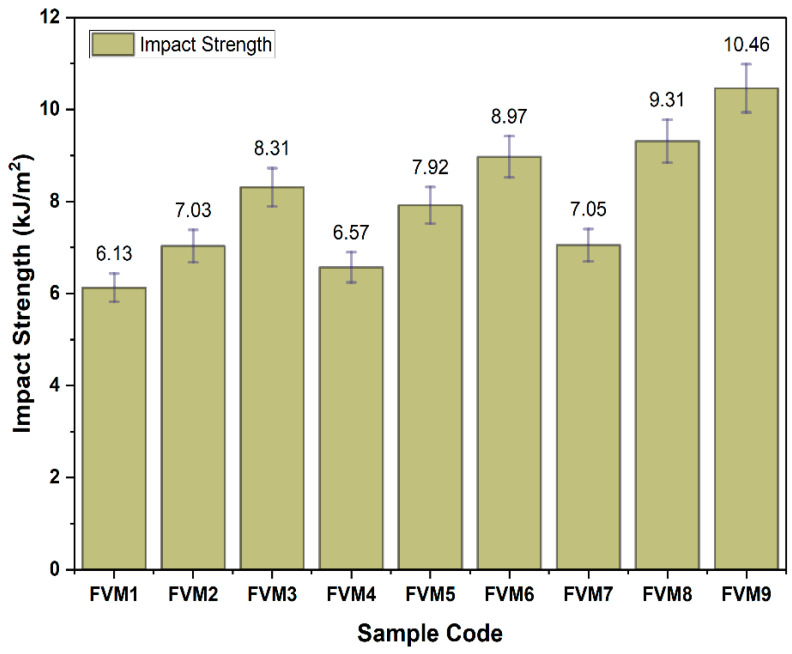
Impact properties of flax/vetiver/MFF (FVM) hybrid composites.

Impact resistance was the highest for FVM9 at 10.46 kJ/m^2^, compared to FVM1’s 6.13 kJ/m^2^ (Figure 10). At the microstructural level, FVM9’s enhanced impact resistance is due to the synergistic interaction between flax and vetiver fibers. The high elongation and toughness of vetiver enable it to absorb impact energy through plastic deformation, whereas the stiffness of flax (73.7% cellulose) maintains structural integrity, preventing brittle failure. The layered structure positions vetiver as the outer skin, allowing it to dissipate the initial impact energy, while the flax core resists crack propagation, as the denser fiber network (20% each) minimizes void content and enhances energy dissipation pathways.

Alkali treatment improves interfacial adhesion by reducing the amount of amorphous components (e.g., vetiver hemicellulose, which decreases from 39.8% to 22.5%) and decreasing the number of weak interfacial regions that could fail under dynamic loading. In MFF-inclusive samples, such as FVM6 (8.97 kJ/m^2^) and FVM8 (9.31 kJ/m^2^), the fine particles of the filler (33.75 µm) increased the surface area for adhesion, enhancing energy transfer and reducing microcrack formation. However, excessive MFF can lead to agglomeration, creating stress concentration points that lower impact resistance (e.g., FVM3, 7.25 kJ/m^2^). FVM1’s poor performance is attributed to its sparse fiber network (10% each), which increases void content and weakens the interfacial bond, leading to rapid crack propagation under impact. The lack of sufficient fiber reinforcement fails to provide adequate energy dissipation mechanisms, resulting in a brittle failure at the microstructural level.

### 3.4. Overall Properties of Mechanical Properties

**Figure 11:**
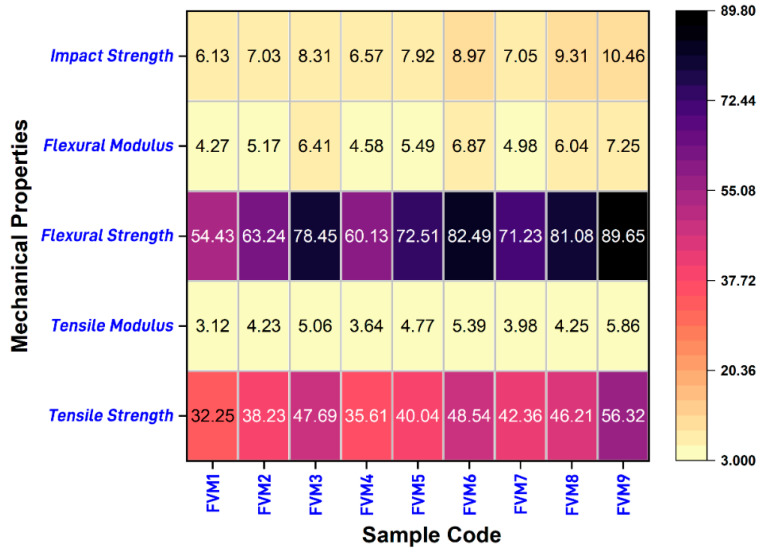
Preference order of mechanical properties for FVM hybrid composites.

The overall mechanical performance of the FVM composites is governed by the microstructural interactions between flax, vetiver, and MFF, which influence the fiber distribution, void content, and interfacial adhesion within the epoxy matrix (Figure 11). In high-performing samples such as FVM9, the balanced fiber content (20% flax, 20% vetiver) ensures a dense fiber network that minimizes voids and enhances stress transfer, while the flax–vetiver synergy optimizes stiffness and toughness, improving the tensile, flexural, and impact properties.

Alkali treatment plays a critical role in increasing cellulose content (e.g., MFF: 37.4% to 52.5%) and reducing moisture, which strengthens the fiber–matrix interface, reducing the likelihood of fiber pull-out or delamination. In MFF-inclusive samples (e.g., FVM6 and FVM8), the filler improved matrix cohesion by filling microvoids, although agglomeration occurred at higher contents (>10 wt.%) introduces stress concentration points, deteriorating performance by initiating micro-cracks. Lower-performing samples, such as FVM1, suffer from sparse fiber networks, higher void fractions, and weaker interfacial bonding, leading to stress concentrations and rapid crack propagation at the microstructural level. These findings highlight the importance of optimizing the fiber ratios and filler content to achieve a uniform microstructure, suggesting that future studies could employ SEM analysis to directly visualize the fiber distribution and void content, further elucidating the microstructural mechanisms driving mechanical performance.

### 3.5. Comparison with the Similar Work

The comparison table (Table 5) includes various matrix types (epoxy, PLA, polyester, etc.), focusing on tensile strength, flexural strength, impact resistance, filler wt.%, and overall density.

Compared with all of the similar studies, this flax/vetiver/MFF hybrid composite stands out by coupling high specific strength (~56 MPa tensile, ~89 MPa flexural) with reduced density, estimated at 1.05–1.15 g/cm^3^, making it lighter than many flax-based systems and significantly lighter than composites with traditional fillers like calcium carbonate or glass fibers. This positions it as a promising material for lightweight structural applications where the strength-to-weight ratio is critical.

## 4. CRITIC—EDAS Optimization

In this study, the CRITIC and EDAS MCDM methods were employed to assess and rank the mechanical properties of FVM hybrid composites. The CRITIC method was used to determine the weights of tensile, flexural, and impact strengths, whereas the EDAS method was applied to rank the composite variants based on these properties. In addition, comparisons with WASPAS, COPRAS, TOPSIS, and VIKOR methods were conducted to validate the reliability of the rankings. The influence of weight variation on the final rankings was analyzed through a sensitivity analysis.

The CRITIC method quantifies the relative importance of each criterion (tensile, flexural, and impact strengths) using contrast intensity (Table S1) and inter-criteria correlations (Table 6) as follows: The derived weights (0.3195 for tensile, 0.3426 for flexural, and 0.3377 for impact) highlight the significant contributions of all three properties to the overall composite performance (Table 6). These weights ensure an unbiased evaluation of the composite variants in subsequent analyses.

The EDAS method was used to rank the FVM composites. Initially, the average solution (Δ_j_) for each criterion was computed (38.364 MPa for tensile, 65.352 MPa for flexural, and 7.192 kJ/m^2^ for impact), as shown in Table 7. Positive Distance from the Average (PDA) and Negative Distance from the Average (NDA) values were calculated for each composite, considering all criteria as beneficial (Table S2). These distances were weighted using CRITIC-derived weights, yielding SP_i_ (Weighted PDA) and SN_i_ (Weighted NDA), as presented in Table S3.

Normalized weighted sums (NSP_i_ and NSN_i_) were calculated to standardize the distances across all alternatives (Table 8). The final appraisal scores (Ø_i_) were determined by averaging NSP_i_ and NSN_i_ scores. FVM9 emerged as the top-performing composite with a perfect Ø_i_ score of 1, whereas FVM1 ranked the lowest with a score of zero. These results are consistent with the mechanical testing results, validating the robustness of the EDAS method for ranking composite materials.

**Figure 12:**
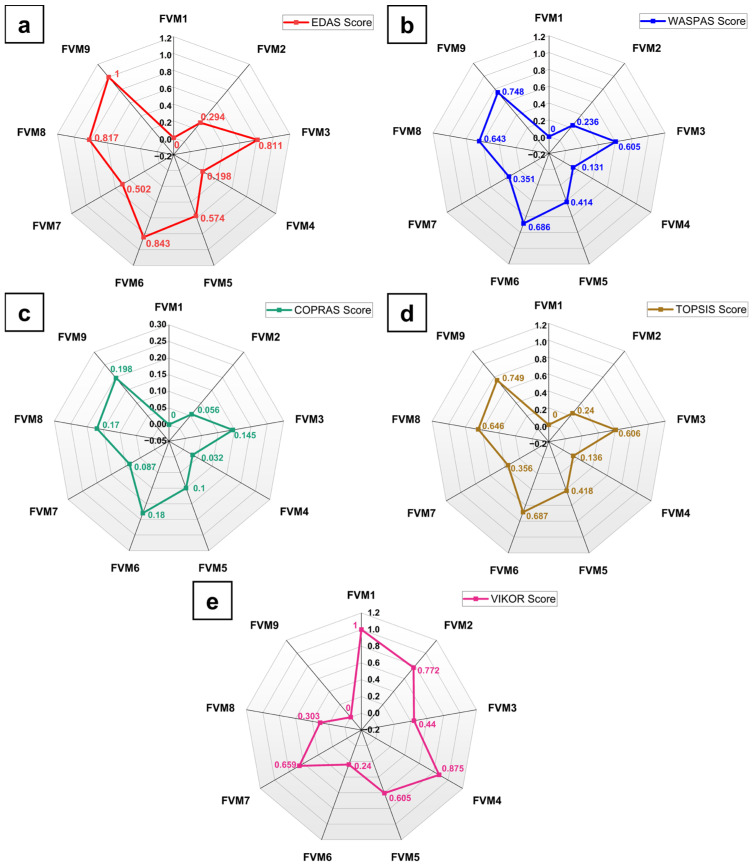
Comparison of (**a**) EDAS score with (**b**) WASPAS, (**c**) COPRAS, (**d**) TOPSIS, and (**e**) VIKOR.

To ensure the reliability of the rankings, alternative MCDM methods, including WASPAS, COPRAS, TOPSIS, and VIKOR, were applied to the dataset. Figure 12 and Table S4 present the scores and ranks obtained using each method, respectively. The rankings produced by all methods showed consistent top performance for FVM9, followed by FVM6, and FVM8. The near-unanimity of the top-ranking alternatives across methods demonstrates the robustness and reliability of the CRITIC–EDAS approach.

The consistent top ranking of FVM9 highlights the synergistic effects of flax and vetiver fibers combined with optimized filler content. Flax fibers, with their high tensile strength, provide a rigid core, whereas vetiver fibers contribute energy-absorbing properties. The addition of MFF enhanced interfacial bonding and stress transfer within the epoxy matrix, resulting in superior mechanical performance. The CRITIC–EDAS methodology provides an objective framework for evaluating and ranking composite materials. By incorporating variability and inter-criteria relationships, the CRITIC method ensures unbiased weight determination. The subsequent application of the EDAS effectively combined these weights with performance measures to systematically rank the composites. This approach not only validates the experimental findings but also facilitates informed decision-making for material optimization.

### 4.1. Optimality Criterion for Selecting FVM Hybrid Composite Composition

**Figure 13:**
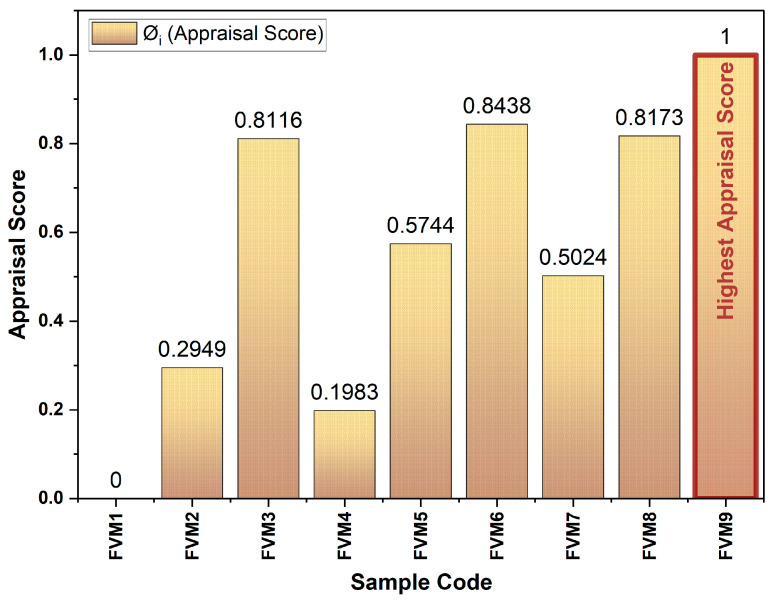
Optimality criterion based on appraisal score.

The optimality criterion for selecting the composition of the flax/vetiver/mahogany fruit filler (FVM) hybrid composite was to maximize the mechanical performance, as determined by the highest combined appraisal score derived from the CRITIC–EDAS method. This score integrates weighted evaluations of tensile strength, flexural strength, and impact resistance, with weights assigned based on the importance of the criteria and inter-criteria correlations (0.3195 for tensile, 0.3426 for flexural, and 0.3377 for impact). The graph (Figure 13) accurately represents the appraisal scores from Table 7, with FVM9 as the top performer (Øi = 1), followed by FVM6 (0.8438) and FVM8 (0.8173), which aligns with the document’s findings on mechanical performance. The optimal composition also considers the synergistic effects of flax and vetiver fibers with MFF reinforcement, enhanced interfacial bonding through alkali treatment, and application-specific requirements, such as lightweight properties for automotive, construction, aerospace, and marine applications.

### 4.2. Sensitivity Analysis

Sensitivity analysis was used to evaluate the impact of varying the criterion weights on the rankings. Two alternative weight scenarios (S1 and S2) were compared with the actual conditions of the study.

**S1**: The weights were adjusted to 0.3426, 0.3377, and 0.3195 for the tensile, flexural, and impact tests, respectively.

**S2**: The weights were adjusted to 0.3377, 0.3195, and 0.3426 for the tensile, flexural, and impact tests, respectively.

Table 9 presents the Ø_i_ scores and rankings for all scenarios. Despite the changes in weights, the top three composites (FVM9, FVM6, and FVM8) retained their positions across all scenarios, indicating negligible sensitivity to weight variations. This robustness underscores the reliability of the CRITIC–EDAS method in assessing composite performance. The integration of the CRITIC and EDAS methods successfully ranked the mechanical performance of the FVM hybrid composites and identified FVM9 as the optimal composition. Comparisons with other MCDM methods confirmed the reliability of these rankings. Sensitivity analysis demonstrated a minimal impact of weight variation on ranking stability. These findings underscore the utility of CRITIC–EDAS for optimizing composite materials and highlight the potential of FVM composites for high-performance applications across various industries.

The selection of MFFs in the composite formulations, despite top compositions such as FVM9 not containing MFFs, is justified by their ability to provide lightweight properties and comparable strength, which aligns with application-specific needs. FVM6 (15% vetiver, 20% MFF, 10% flax) and FVM8 (20% vetiver, 15% MFF, 5% flax) incorporated MFF, yielding good properties next to the best composition (FVM9). Although these values are lower than those of FVM9, MFF-based compositions (FVM6 and FVM8) are closer to the top priority in terms of strength (a difference in only ~8%), while offering a significant advantage in lightweight properties owing to mahogany’s lower density (0.55 g/cm^3^) compared to other fillers, such as flax. This balance makes MFF a practical choice for applications where weight reduction is critical without substantially compromising strength, such as automotive panels or aerospace interiors, where lightweight materials reduce fuel consumption while maintaining structural integrity. The experiment demonstrated that composition selection can be tailored for specific applications. For instance, FVM9’s high strength suits heavy-duty structural components, whereas FVM6 and FVM8, with MFF, are better for lightweight applications, such as portable furniture or boat interiors, where mechanical strength is sufficient and reduced weight enhances functionality. This application-based approach highlights the strategic use of MFF to balance mechanical performance and weight, ensuring optimal material selection for diverse engineering needs.

### 4.3. Limitations and Future Scope of the Present Study

The investigation of flax/vetiver/mahogany fruit filler (FVM) hybrid composites yielded promising results, opening several exciting avenues for future research. Based on the current study, which identified 10 wt.% MFF as optimal through the CRITIC–EDAS method, future work can explore higher concentrations (e.g., 15 or 20 wt.%) to determine the precise threshold for optimal mechanical performance, potentially uncovering new configurations that further enhance tensile, flexural, and impact properties. Investigating the performance of composites under diverse environmental conditions, such as humidity, temperature extremes, or UV exposure, will ensure their reliability for real-world applications in the construction, marine, and aerospace industries. Furthermore, incorporating advanced statistical analyses to quantify the variability in fiber quality and alignment, conducting practical application testing through prototypes (e.g., automotive panels and boat interiors), and tailoring the CRITIC–EDAS method’s criteria weights for specific applications will enhance the industrial relevance of the composites. Finally, future studies should focus on scaling up the fabrication process and optimizing the cost-effectiveness of using flax, vetiver, and MFF, particularly by streamlining the alkali treatment process, to facilitate broader adoption in sustainable engineering solutions. These research directions will further unlock the potential of FVM hybrid composites, ensuring their successful integration into diverse industrial applications in the future.

## 5. Conclusions

The investigation of flax/vetiver/mahogany fruit filler (MFF) hybrid composites demonstrates their potential as high-performance, sustainable materials for industrial applications, with FVM9 (20% flax, 20% vetiver, 0% MFF) emerging as the optimal configuration through the CRITIC–EDAS method, achieving a tensile strength of 56.32 MPa, flexural strength of 89.65 MPa, and impact resistance of 10.46 kJ/m^2^, with a perfect appraisal score of 1.00. Alkali treatment of MFF, vetiver, and flax fibers significantly enhanced interfacial bonding, as evidenced by FTIR, which showed increased cellulose content (37.4% to 52.5% in MFF) and reduced hemicellulose and lignin. XRD confirmed an increase in the crystallinity index from 86.67% to 90.07% and crystalline size growth from 8.10 nm to 8.99 nm, indicating improved structural order and stiffness. Particle size analysis via ImageJ further revealed a refined mean particle size of 33.75 µm in treated MFF, enhancing fiber-matrix adhesion and mechanical consistency. The synergistic effects of flax’s tensile strength, vetiver’s energy absorption, and MFF’s lightweight properties (density 0.55 g/cm^3^) make these composites ideal for applications in the automotive, construction, and aerospace sectors, where FVM9 suits heavy-duty structural components, and MFF-inclusive variants such as FVM6 and FVM8 (with strengths ~8% lower than FVM9) cater to lightweight needs such as portable furniture or boat interiors, balancing performance and weight reduction for sustainability and cost-effectiveness. The robustness of the CRITIC–EDAS rankings, validated by WASPAS, COPRAS, TOPSIS, and VIKOR and supported by sensitivity analysis, underscores the reliability of this approach, paving the way for tailored material optimization in eco-friendly engineering solutions.

## Figures and Tables

**Table 1:** Chemical compositions of mahogany fruit fillers (MFFs).

Factors	Cellulose (%)	Hemicellulose (%)	Lignin (%)	Ash (%)	Moisture Content (%)
Before 5% NaOH treatment	37.4	18.2	23.5	6.8	11.1
After 5% NaOH treatment	52.5	10.3	13.1	4.3	7.6

**Table 2:** Chemical compositions of vetiver and flax fibers.

Factors	Cellulose (%)	Hemicellulose (%)	Lignin (%)	Ash (%)	Moisture Content (%)
**Vetiver Fibers**					
Before 5% NaOH treatment	32.3	39.8	11.2	4.3	12.4
After 5% NaOH treatment	48.7	22.5	6.8	2.5	8.3
**Flax Fibers**					
Before 5% NaOH treatment	62.6	16.1	3.4	3.9	9.3
After 5% NaOH treatment	73.7	9.5	2.3	1.5	6.2

**Table 3:** Process control factors.

Factors	Flax Fiber (wt.%)	Vetiver Fiber (wt.%)	MFF (wt.%)
Flax fiber mat (wt.%)	10	15	20
Unidirectional vetiver fiber (wt.%)	10	15	20
Mahogany fruit filler (MFF) (wt.%)	0	5	10

**Table 4:** Taguchi orthogonal array.

Table Trial Run	Sample Code	Flax Fiber (wt.%)	Vetiver Fiber (wt.%)	MFF (wt.%)
1	FVM1	10	10	0
2	FVM2	10	15	10
3	FVM3	10	20	5
4	FVM4	15	10	5
5	FVM5	15	15	0
6	FVM6	15	20	10
7	FVM7	20	10	10
8	FVM8	20	15	5
9	FVM9	20	20	0

**Table 5:** Comparison of MFF filled composites with other studies.

Composite System	Filler wt.%	Matrix	Fibers	Fillers	Density (g/cm^3^)	References
Flax/Vetiver + Mahogany Fruit Filler (MFF)	10	Epoxy	Flax + Vetiver	MFF	1.05–1.15	Present Work
Flax + Wheat Bran	6	Epoxy	Flax	Wheat Bran	1.25–1.30	[52]
Luffa Fiber + Wood Dust	20	Phenol–formaldehyde	Luffa acutangula	Wood Dust	1.10–1.30	[53]
Jute Fabric + Eggshell	10	Polyester	Jute fabric	Eggshell	1.20–1.30	[54]
Flax Fiber + Bran Filler	Variable	Epoxy	Flax	Bran	1.25	[55]
Hybrid Jute + Jack Tree + Eggshell	10	Polyester	Jute + Jack tree	Eggshell	1.25–1.35	[56]
Jute Fiber + Eggshell + Nanoclay	3–12	Epoxy	Jute fiber	Nanoclay/Eggshell	1.25–1.30	[57]
Luffa + Coir + Wood Dust	10	Phenolic	Luffa, Coir	Wood Dust	1.1–1.3	[58]
Wheat Fiber + Bran Filler	10	Epoxy	Wheat fiber	Bran	1.20–1.30	[59]
Glass–Jute Hybrid + Eggshell Ash	10	Polyester	Glass + Jute	Eggshell Ash	1.5–1.6	[60]
Jute + Eggshell	12	Epoxy	Jute	Eggshell	1.45	[61]

**Table 6:** Correlation coefficient and calculated weightage.

	Tensile Strength (MPa)	Flexural Strength (MPa)	Impact Strength (kJ/m^2^)	CCj	OWj
**Tensile (MPa)**	1	0.9735	0.944578	0.5926	0.3195
**Flexural (MPa)**	0.9735	1	0.9623	0.6356	0.3426
**Impact (kJ/m^2^)**	0.9445	0.9623	1	0.6265	0.3377

**Table 7:** Average solution of each criterion in the EDAS method.

Criterion	Average Solution (Δ_j_)
Tensile Strength (MPa)	38.364
Flexural Strength (MPa)	65.352
Impact Strength (kJ/m^2^)	7.192

**Table 8:** Normalized sum of PDA and NDA values and appraisal score of alternatives.

Sample Code	NSP_i_ (Normalized PDA)	NSN_i_ (Normalized NDA)	Ø_i_ (Appraisal Score)	Rank
FVM1	0	0	0	9
FVM2	0	0.5897	0.2949	7
FVM3	0.6232	1	0.8116	4
FVM4	0	0.3967	0.1983	8
FVM5	0.1489	1	0.5744	5
FVM6	0.6877	1	0.8438	2
FVM7	0.1921	0.8127	0.5024	6
FVM8	0.6345	1	0.8173	3
FVM9	1	1	1	1

**Table 9:** Sensitivity analysis.

	R_i_								
	FVM1	FVM2	FVM3	FVM4	FVM5	FVM6	FVM7	FVM8	FVM9
Actual Condition	0	0.254	0.632	0.143	0.450	0.727	0.392	0.713	1
S1	0	0.255	0.634	0.144	0.447	0.727	0.396	0.709	1
S2	0	0.254	0.632	0.143	0.447	0.725	0.391	0.711	1
Ranking	FVM9 > FVM 6 > FVM 8 > FVM 3 > FVM 5 > FVM 7 > FVM 2 > FVM 4 > FVM 1								

## Data Availability

The original contributions presented in this study are included in the article/Supplementary Material. Further inquiries can be directed to the corresponding author.

## Supplementary Materials

The following supporting information can be downloaded at: https://www.mdpi.com/article/10.3390/polym17131790/s1, Table S1: Normalized decision matrix obtained using the CRITIC approach; Table S2: PDA and NDA values of criteria; Table S3: Weighted sum of PDA and NDA values of criteria; Table S4: Comparison of results with other techniques.

## Abbreviations

The following abbreviations are used in this manuscript:

CRITIC	Criteria Importance Through Inter-Criteria Correlation
EDAS	Evaluation based on Distance from Average Solution
FVM	Flax/Vetiver/Mahogany Fruit Filler (Hybrid Composite)
MFF	Mahogany Fruit Filler
FTIR	Fourier Transform Infrared Spectroscopy
XRD	X-Ray Diffraction
SEM	Scanning Electron Microscopy
MCDM	Multi-criteria decision-making
WASPAS	Weighted Aggregated Sum Product Assessment
COPRAS	Complex Proportional Assessment
TOPSIS	Technique for Order Preference by Similarity to Ideal Solution
VIKOR	VlseKriterijumska Optimizacija I Kompromisno Resenje
DoE	Design of Experiments
UTM	Universal Testing Machine
PDA	Positive Distance from Average
NDA	Negative Distance from Average
NSP	Normalized Sum of Positive Distance
NSN	Normalized Sum of Negative Distance
PLA	Polylactic Acid
MABAC	Multi-Attributive Border Approximation Area Comparison
CrI	Crystallinity Index
NaOH	Sodium Hydroxide
PVA	Polyvinyl Alcohol
