# Supplementary material for: CRITIC–EDAS Approach for Evaluating Mechanical Properties of Flax/Vetiver/MFF Hybrid Composites
Source: Polymers (Basel). 2025 Jun 27;17(13):1790. doi: 10.3390/polym17131790 (PMC12252127; doi:10.3390/polym17131790)
Supplement: Supplementary file 1 [file polymers-17-01790-s001.zip › polymers-3684634-supplementary.pdf]

CRITIC-EDAS Approach for Evaluating Mechanical Properties of Flax/Vetiver/MFF Hybrid Composites

Navin M [1,*], Ramakrishnan Thirumalaisamy [2], Balaji Devarajan [3] and Bhuvaneswari Venkateswaran [3,4]

[1] Department of Mechanical Engineering, CMS College of Engineering and Technology, Coimbatore 641032, Tamil Nadu, India
[2] Department of Mechanical Engineering, Sri Eshwar College of Engineering, Coimbatore 641202, Tamil Nadu, India; ramakrishnan.t@sece.ac.in
[3] Department of Mechanical Engineering, KPR Institute of Engineering and Technology, Coimbatore 641407, Tamil Nadu, India; balaji.ntu@gmail.com
[4] AU-Sophisticated Testing and Instrumentation Centre and Department of Mechanical Engineering, Alliance School of Applied Engineering, Alliance University, Bengaluru 562106, Karnataka, India; bhuvanashankar82@gmail.com

* Correspondence: navinarok@gmail.com

CRITIC Method:

Step 1:

Develop a decision matrix by organizing the performance values of all response parameters. This matrix is expressed as shown in **Equation (1)**.

$$C = [c_{ij}]_{n\times m} = \begin{bmatrix} c_{11} & c_{12} & \cdots & c_{1m} \\ c_{21} & c_{22} & \cdots & c_{2m} \\ \vdots & \vdots & \ddots & \vdots \\ c_{n1} & c_{n2} & \cdots & c_{nm} \end{bmatrix} \quad (1)$$

Here, c_{ij} represents the performance data for the jth response corresponding to the ith design alternative.

Step 2:

The decision matrix is normalized to eliminate the influence of different units or scales using **Equation (2)**:

$$c'_{ij} = \frac{c_{ij} - \min(c_{ij})}{\max(c_{ij}) - \min(c_{ij})} \tag{2}$$

where c'_{ij} denotes the normalized performance values.

Step 3:

The correlation coefficients between the criteria were determined to quantify the interrelationships. This is calculated using **Equation (3),**

$$CC_j = \sum_{j^*=1}^{n} (1 - r_{jj^*}) \tag{3}$$

where r_{jj^*} represents the correlation coefficient between the jth and j^{*th} criteria.

Step 4:

Compute the objective weight for each criterion using the correlation values from Step 3 using **Equation (4).**

$$OW_j = \frac{CC_j}{\sum_{j=1}^{n} CC_j} \tag{4}$$

These steps ensure the systematic evaluation of criteria weighting, accounting for both variability and interdependencies in the decision-making process.

EDAS Method

Step 1: Calculate the average solution (Δ_j) for each criterion from the decision matrix using **Equation (5)**.

$$\Delta_j = \frac{\sum_{i=1}^{n} C_{ij}}{n}, j = 1,2,\ldots,n \tag{5}$$

where C_{ij} denotes the value of the ith alternative for the jth criterion.

Step 2: Determine the Positive Distance from the Average (PDA) and Negative Distance from the Average (NDA) for each criterion, based on whether the criterion is beneficial or non-beneficial. All criteria in this study were assumed to be beneficial, and PDA and NDA were calculated using **Equation (6) and (7)** respectively:

$$PDA_{ij} = \begin{cases} max\left(0, \frac{C_{ij} - \Delta_j}{\Delta_j}\right) \ if\ the\ creterion\ is\ beneficial \\ max\left(0, \frac{\Delta_j - C_{ij}}{\Delta_j}\right) \ if\ the\ criterion\ is\ non-beneficial \end{cases} \tag{6}$$

$$NDA_{ij} = \begin{cases} max\left(0, \frac{\Delta_j - C_{ij}}{\Delta_j}\right) \ if\ the\ creterion\ is\ beneficial \\ max\left(0, \frac{C_{ij} - \Delta_j}{\Delta_j}\right) \ if\ the\ criterion\ is\ non-beneficial \end{cases} \tag{7}$$

Step 3: Compute the weighted sum of PDA (SP_i) and NDA (SN_i) for each alternative using the assigned weights (w_j) for each criterion by **Equation (8),**

$$\begin{aligned} SP_i &= \sum\nolimits_{j=1}^{m} w_j \times PDA_{ij} \\ SN_i &= \sum\nolimits_{j=1}^{n} w_j \times NDA_{ij} \end{aligned} \tag{8}$$

Step 4: Normalize the weighted sums of PDA (NSP_i) and NDA (NSN_i) using **Equation (9),**

$$\begin{aligned} NSP_i &= \frac{SP_i}{\max_i(SP_i)} \\ NSN_i &= 1 - \frac{SN_i}{\max_i(SN_i)} \end{aligned} \tag{9}$$

Step 5: Calculate the appraisal score (ϕ_i) for each alternative by averaging the normalized values of PDA and NDA using **Equation (10).**

$$\phi_i = \frac{NSP_i + NSN_i}{2} \tag{10}$$

This score was used to rank the alternatives, with higher scores indicating an optimal MFF concentration. These steps allow for the systematic and structured evaluation of alternatives using the EDAS methodology.

Table S1: Normalized decision matrix obtained using the CRITIC approach

Sample Code	Tensile (MPa)	Flexural (MPa)	Impact (kJ/m^2)
FVM1	0	0	0
FVM2	0.2484	0.2501	0.2078
FVM3	0.6414	0.6819	0.5034
FVM4	0.1395	0.1618	0.1016
FVM5	0.3236	0.5133	0.4133
FVM6	0.6767	0.7967	0.6558
FVM7	0.4200	0.4770	0.2124
FVM8	0.5799	0.7566	0.7344
FVM9	1	1	1

Table S2: PDA and NDA values of criteria

Sample Code	Tensile (PDA)	Flexural (PDA)	Impact (PDA)	Tensile (NDA)	Flexural (NDA)	Impact (NDA)
FVM1	0	0	0	0.1943	0.2255	0.2380
FVM2	0	0	0	0.0391	0.0977	0.1391
FVM3	0.2431	0.2007	0.1528	0	0	0
FVM4	0	0	0	0.0978	0.1441	0.1683
FVM5	0.0436	0.1097	0.1022	0	0	0
FVM6	0.2764	0.2616	0.2233	0	0	0
FVM7	0.0673	0.0885	0.0019	0	0	0.1280
FVM8	0.2033	0.2437	0.2880	0	0	0
FVM9	0.4695	0.3711	0.4518	0	0	0

Table S3: Weighted sum of PDA and NDA values of criteria

Sample Code	SPi (Weighted PDA)	SNi (Weighted NDA)
FVM1	0	0.2320
FVM2	0	0.0952
FVM3	0.2347	0
FVM4	0	0.1395
FVM5	0.0561	0
FVM6	0.2589	0
FVM7	0.0720	0.0433
FVM8	0.2389	0
FVM9	0.3766	0

Table S4: Comparison of results with other techniques

Sample Code	EDAS Score	EDAS Rank	WASPAS Score	WASPAS Rank	COPRAS Score	COPRAS Rank	TOPSIS Score	TOPSIS Rank	VIKOR Score	VIKOR Rank
FVM1	0	9	0	9	0	9	0	9	1	9
FVM2	0.294	7	0.236	7	0.056	7	0.240	7	0.772	7
FVM3	0.811	4	0.605	4	0.145	4	0.606	4	0.440	4
FVM4	0.198	8	0.131	8	0.032	8	0.136	8	0.875	8
FVM5	0.574	5	0.414	5	0.100	5	0.418	5	0.605	5
FVM6	0.843	2	0.686	2	0.180	2	0.687	2	0.240	2
FVM7	0.502	6	0.351	6	0.087	6	0.356	6	0.659	6
FVM8	0.817	3	0.643	3	0.170	3	0.646	3	0.303	3
FVM9	1	1	0.748	1	0.198	1	0.749	1	0	1
